# Supplementary material for: Causal Relationship between Aging and Anorexia Nervosa: A White-Matter-Microstructure-Mediated Mendelian Randomization Analysis
Source: Biomedicines. 2024 Aug 16;12(8):1874. doi: 10.3390/biomedicines12081874 (PMC11351342; doi:10.3390/biomedicines12081874)
Supplement: Supplementary file 1 [file biomedicines-12-01874-s001.zip › Supplementary Figure/Figure S7/Single SNP plot of MR analysis for ubm-b-1940 to AN.pdf]

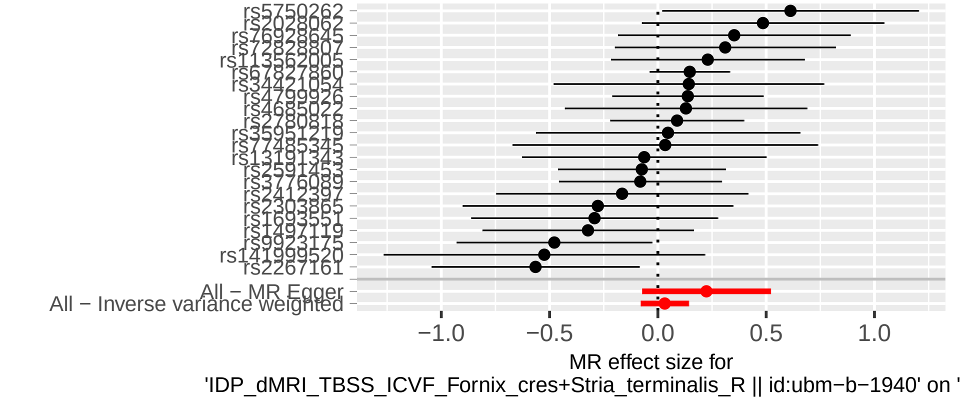
rs5750262
rs2028062
rs76928645
rs72828807
rs113562005
rs67827860
rs34421054
rs4799926
rs4685022
rs2780818
rs35951219
rs77485345
rs13191343
rs2591453
rs3776089
rs2412397
rs2303865
rs1693551
rs1497119
rs9923175
rs141999520
rs2267161
All − MR Egger
All − Inverse variance weighted
−1.0
−0.5
0.0
0.5
1.0
MR effect size for
'IDP_dMRI_TBSS_ICVF_Fornix_cres+Stria_terminalis_R || id:ubm−b−1940' on '
